# Gender Identity and Sexual Orientation Identity in Women and Men Prenatally Exposed to Diethylstilbestrol

**DOI:** 10.1007/s10508-020-01637-7

**Published:** 2020-01-23

**Authors:** Rebecca Troisi, Julie R. Palmer, Elizabeth E. Hatch, William C. Strohsnitter, Dezheng Huo, Marianne Hyer, Karen I. Fredriksen-Goldsen, Robert Hoover, Linda Titus

**Affiliations:** 1 Division of Cancer Epidemiology and Genetics, National Cancer Institute, Bethesda, MD 20892 USA; 2 Slone Epidemiology Center, Boston University, Boston, MA USA; 3 Department of Epidemiology and Biostatistics, Boston University School of Public Health, Boston, MA USA; 4 Department of Molecular, Cell and Cancer Biology, University of Massachusetts Medical School, Worcester, MA USA; 5 Department of Public Health Sciences, University of Chicago, Chicago, IL USA; 6 Information Management Services, Rockville, MD USA; 7 School of Social Work, University of Washington, Seattle, WA USA; 8 Departments of Epidemiology and Pediatrics, Geisel School of Medicine at Dartmouth, Lebanon, NH USA

**Keywords:** Diethylstilbestrol, Sexual orientation identity, Gender identity, In utero exposure

## Abstract

We assessed the associations of prenatal diethylstilbestrol (DES) exposure, a potent estrogen, with sexual orientation and gender identity in 3306 women and 1848 men who participated in a study of prenatal DES exposure. Odds ratios (OR) and 95% confidence intervals (CI) were derived from logistic regression models adjusted for birth year, study cohort, and education. Among women, the OR for DES in relation to reporting sexual orientation identity as nonheterosexual was 0.61 (95% CI 0.40–0.92) primarily due to a strong inverse association with a lesbian identity (OR 0.44, 95% CI 0.25–0.76). Among men, the OR for DES in relation to reporting a nonheterosexual sexual orientation identity was 1.4 (95% CI 0.82–2.4), and ORs were similar for having a gay identity (1.4, 95% CI 0.72–2.85) and bisexual identity (1.4, 95% CI 0.57–3.5). Only five individuals reported a gender identity not conforming to that assigned at birth, preventing meaningful analysis. Women who were prenatally exposed to DES were less likely to have a lesbian or bisexual orientation, while DES-exposed men were somewhat more likely to report being gay or bisexual, but estimates were imprecise.

## Introduction

Research on the biological foundations of sexual orientation, an enduring pattern of emotional, romantic, and/or sexual attractions to men, women, or both sexes (American Psychological Association, 2019) supports roles for genetics, epigenetics, and prenatal hormonal exposure, in particular testosterone (Berenbaum & Beltz, 2016; Hines, 2011a, 2020; Fisher, Ristori, Morelli, & Maggi, 2018). The origins of gender identity, a person’s concept of self as female, male, or other, may be more complex regarding the relative contributions of social and biological influences and the role of early testosterone exposure (Berenbaum & Beltz, 2016; Hines, 2011b).

In general, experimental studies in animals show that high concentrations of testosterone at critical developmental periods increase male-typical neural and behavioral development while lower concentrations result in female-typical neural and behavioral development (reviewed in Hines, 2011a). Direct data in humans on sexual orientation are based on studies of rare conditions associated with an altered hormonal milieu in utero. Classical congenital adrenal hyperplasia (CAH) is an autosomal recessive disorder involving prenatal androgen excess that can result in virilization of the external genitalia in females. Results of numerous studies of women with CAH show that these women are less likely to be exclusively or almost exclusively heterosexual compared with women who do not have CAH, and some of these studies show a positive association with severity of the disorder (Hines, 2011a). In contrast, most studies in men with CAH find little difference in sexual orientation (Gehrmann et al., 2019). Androgen insensitivity syndrome (AIS) is an intersex condition that results in partial or complete inability of the cell to respond to androgens. Of the few studies on complete AIS, when the external genitalia are female, most find that these individuals tend to have an androphilic sexual orientation (Hines, Ahmed, & Hughes, 2003; Wisniewski et al., 2000).

The use of diethylstilbestrol, a synthetic nonsteroidal estrogen, during pregnancy, provides another test of the influence of prenatal hormonal exposure on sexual orientation. Despite its lack of efficacy, high-dose exposure occurred when DES was administered to several million pregnant women in the U.S. and Europe from the early 1940s through the 1960s (Noller & Fish, 1974) for prevention of spontaneous abortion and premature delivery. With the demonstration in 1971 of a strong association between DES and clear cell adenocarcinoma of the vagina and cervix in young women (Herbst, Ulfelder, & Poskanzer, 1971), use in pregnancy was discontinued.

DES is a highly potent full agonist of the nuclear estrogen receptors, estrogen receptor (ER)α and ERβ (Jordan, 2013; Seiler, Autrup, & Autrup, 2012), and compared to estradiol, DES has greater affinity to ERs (Kuiper et al., 1997) and bioavailability when taken orally. DES can be metabolized in all species evaluated to produce either hormonally inactive or estrogenic compounds (Korach, Metzler, & McLachlan, 1978), but metabolism in human pregnancy is unknown.

Prenatal exposure to DES from rodent studies shows increased estrogen, progesterone, and testosterone production in females owing to enlargement of the ovary, though even with correction for ovarian size testosterone production remains elevated in the exposed (Haney, Newbold, & McLachlan, 1984). In male rodents, relatively low-dose exposure does not affect steroidogenesis; however, higher doses of DES suppress testicular testosterone production as well as androgen receptor expression (Adamsson, Brokken, Paranko, & Toppari, 2008). While we do not know how DES was metabolized in human pregnancy, outcomes in animals exposed to DES during development have been extremely like those in comparably exposed humans (McLachlan, 2016).

Based on the animal paradigm and human evidence indicating that prenatal testosterone exposure influences male-type development, DES could influence nonheterosexual orientation in women by increasing testosterone production, a phenomenon that has been observed in female mice exposed in utero to DES (Haney et al., 1984). Alternatively, rapid metabolism of testosterone to estradiol in normal human pregnancy may protect the female fetus from androgen effects. In males, high doses of DES could affect nonheterosexual orientation by decreasing testosterone production and/or androgen receptor expression.

While a review of gender-related behavior in women exposed prenatally to DES compared with women who were unexposed concluded that there were no distinct differences in gender-related behaviors between exposed and unexposed women (Newbold, 1993), there was some evidence for greater prevalence of certain sexual characteristics, including bisexuality or homosexuality. We previously reported that prenatally DES-exposed women were less likely than unexposed women to report same-sex partners (odds ratio [OR] = 0.71, 95% confidence interval [CI] 0.51–0.98), but prenatally DES-exposed men were slightly more likely to report same-sex partners than unexposed men (OR = 1.3, 95% CI 0.80–2.1) (Titus-Ernstoff et al., 2003).

Research on sexual orientation and gender identity has markedly advanced since our initial report of usual sexual partners in adulthood based on data collected in 1994. Recently, we ascertained additional information in our cohort on sexual orientation identity, a person’s identity based on their attractions, related behaviors, and membership in a community of others who share those attractions and asked about gender identity for the first time to assess possible associations with prenatal exposure to DES.

## Method

### Participants

The present study used data from a longitudinal cohort study. In 1994, the National Cancer Institute (NCI) combined cohorts that were followed previously (1970s–1980s) and assembled new cohorts (comprising mothers or offspring of existing cohort members) to establish the US NCI DES combined cohort follow-up study. The resulting combined cohort study is comprised of prenatally exposed and unexposed women and men whose mothers attended a large, private infertility practice (Horne Cohort), or participated in the Women’s Health Study of women who were and were not given DES during pregnancy (WHS Cohort; Greenberg et al., 1984), or enrolled in a clinical trial of DES (or placebo) use in pregnancy (Dieckmann Cohort; Bibbo et al., 1977). The women’s combined cohort additionally included prenatally DES-exposed and unexposed women who participated in the National Cooperative Diethylstilbestrol Adenosis Project ([DESAD] cohort; Labarthe et al., 1978); in this cohort, the unexposed were either sisters of the exposed participants (24%) or women identified from the same record review as the exposed (76%). The primary goal of the DESAD study was to provide annual clinical examinations for the daughters to ascertain their risk of vaginal and cervical abnormalities and follow the natural history of these conditions. Because of this, many more exposed than unexposed daughters were enrolled. The men’s combined cohort additionally included prenatally DES-exposed and unexposed men who participated in a study at the Mayo Clinic (Leary et al., 1984).

The NCI follow-up of the combined cohorts began in 1994 with a mailed questionnaire, and subsequent questionnaires were mailed approximately every 5 years in 1997, 2001, 2006, 2011, and 2016. Among the 6571 women (4474 exposed, 2097 unexposed) approached for participation in 1994, 88% of exposed and 84% of unexposed women completed the NCI combined cohort baseline questionnaire. In subsequent follow-up of the NCI combined cohort, participation percents in the exposed and unexposed women were 94% and 94% for the 1997 questionnaire, 90% and 90% for the 2001 questionnaire, 82% and 85% for the 2006 questionnaire, 76% and 77% for the 2011 questionnaire, and 73% and 74% for the 2016 questionnaire, respectively, for the exposed and unexposed.

Among the 3600 men (1710 exposed, 1890 unexposed) approached in 1994, 79% and 72% of exposed and unexposed men completed the NCI combined cohort baseline questionnaire. In subsequent follow-up of the NCI combined cohort, participation percents were 94% and 95% for the 1997 questionnaire, 88% and 86% for the 2001 questionnaire, 83% and 83% for the 2006 questionnaire, 74% and 74% for the 2011 questionnaire, and 75% and 73% for the 2016 questionnaire, respectively, for the exposed and unexposed men.

Eligibility for the current analysis required completion of the questions on sexual orientation identity and gender identity contained in the 2016 follow-up questionnaire. Over the course of the NCI combined cohort study, 6048 women had responded to at least one questionnaire and were considered study participants. One of the women’s subcohorts was not approached in 2016 (*n* = 1007), and an additional 222 women had died since 1994. Of the remaining 4819 women (3223 exposed, 1596 unexposed), 3371 (2260 exposed, 1111 unexposed) agreed to complete the full questionnaire. Of these, 3306 (2220 [98%] and 1086 [98%] of the exposed and unexposed, respectively) answered the relevant questions on sexual orientation identity and gender identity.

Over the course of the NCI combined cohort study, 2924 men had responded to at least one questionnaire and were considered participants; 256 of these had died since 1994. Of the remaining 2668 (1322 exposed, 1346 unexposed) men, 1885 (947 exposed, 938 unexposed) agreed to complete the full 2016 questionnaire. Of these, 1848 (933 [98.5%] exposed and 915 [97.5%] unexposed) answered the relevant questions on sexual orientation identity and gender identity.

### Measures

The 1994 NCI combined cohort baseline questionnaire included a question on the sex of sexual partners in adulthood. In 2016, we included a question on sexual orientation identity (“Which of the following best represents how you think of yourself? Gay or lesbian; bisexual; straight, that is, not gay, lesbian or bisexual; other, please specify; prefer not to respond”) and a question on gender identity (“Which of the following best represents how you currently think of yourself? Woman; man; other, please specify; prefer not to respond”). When participants left the question blank, they were categorized with those who indicated they preferred not to respond. A variable for nonheterosexuality was created by combining responses for gay or lesbian, bisexual, and other. The overlap between those included in the 2016 questionnaire analysis and the 1994 analysis was about 65% for both women and men.

#### DES Exposure

For all combined cohort participants, prenatal exposure to DES, or lack thereof, was documented by the birth record or a physician’s note. Gestational week of first DES exposure was available for 72% of women and 74% of men. We classified the individual cohorts as high or low dose based on differences in prescribing practices by U.S. region. In women, the high-dose cohorts were from Boston (DESAD, WHS subcohort, and Horne), Chicago (Dieckmann), and California (DESAD); low-dose cohorts were from Texas, Minnesota, and Wisconsin (DESAD). In men, the high-dose cohorts were from Boston (WHS subcohort, Horne) and Chicago (Dieckmann); the low-dose cohort was from Mayo Clinic. Dose and gestational age at first DES exposure were unavailable for WHS participants from New Hampshire and Maine, so exposure level could not be classified for those subcohorts (9% of exposed women and 19% of exposed men). Agreement between the dose classification and individual doses was excellent among those with complete data (Palmer et al., 2006). The presence or absence of vaginal epithelial changes (VEC), which are correlated with an earlier and higher DES dose in utero (Herbst, Poskanzer, Robby, Friedlander, & Scully 1975), was available for 99% of prenatally exposed women in the DESAD observational study and the Dieckmann clinical trial cohort but was not available in the other cohorts. Attained education (highest grade completed) was collected on the women’s and men’s 1994 questionnaire.

### Data Analysis

Women and men were analyzed separately. To analyze the 2016 data, OR and 95% CI (used to assess statistical significance) are derived from logistic regression models (Breslow & Day, 1980) with sexual orientation identity (nonheterosexual vs. heterosexual) as the dependent variable, DES exposure as the independent variable, and birth year and cohort as covariates. We additionally included education as a covariate because we thought it might be associated with reporting of sexual orientation identity on the questionnaires. A polytomous logistic regression model was used to model a multinomial response (i.e., a multilevel response whose levels have no inherent ordering) for sexual orientation identity (heterosexual; gay or lesbian; bisexual). In this model, the dependent or outcome variable was dummy coded into multiple 1/0 variables except for one. The model estimated a separate binary logistic regression model for each of the dummy variables with each having its own intercept and regression coefficients (so the predictors can affect each category differently). The association of DES and sexual orientation identity could not be computed separately by cohort because of the small number of outcomes. Associations were evaluated by DES dose (high dose and low dose vs. unexposed as the reference group) and timing of first DES administration (< 8, 8–14, 15 + weeks, missing vs. unexposed as the reference group), and in the Dieckmann and DESAD cohorts, by the presence or absence of VEC in the women (vs. unexposed as the reference group). Regression models for DES dose did not include cohort as a covariate because dose was based on cohort membership. The number of participants reporting a gender identity different from that assigned at birth was too small to analyze statistically. Finally, we compared consistency of participants’ responses to the 2016 question assessing sexual orientation identity with their previous responses to the 1994 question assessing usual sex partners in adulthood.

## Results

The mean age of DES-exposed women was 62.1 years (median = 63.3, SD = 5.4) and of unexposed women was 63.2 years (median = 64.2, SD = 6.3), while in the men the means were 62.9 years (median = 64.8, SD = 6.9) in the exposed and 63.4 years (median = 64.6, SD = 6.1) in the unexposed. Attained education was high overall and higher for the DES-exposed than for the unexposed women and men (Table 1). Study participants were predominantly Caucasian (98% and 97% of exposed and unexposed women and 93% and 92%, respectively, of men).

**Table 1:** Distribution (%) of demographic factors by DES exposure status in women and men

	Women		Men	
	DES exposed	DES unexposed	DES exposed	DES unexposed
	*N* = 2220	*N* = 1086	*N* = 933	*N* = 915
Birth year				
< 1952	36.0	42.6	52.6	47.8
1952–1955	31.0	29.5	20.8	26.6
1956 +	33.0	27.9	26.6	25.7
Cohort				
DESAD	76.3	43.7	–	–
Mayo	–	–	45.7	44.4
Dieckmann	7.6	13.6	15.4	13.9
Horne	6.9	9.3	19.6	11.5
WHS	9.2	33.4	19.3	30.3
Education (from 1994 questionnaire)				
≤ High school (HS)	12.0	16.8	14.7	18.9
Some post-HS	21.2	23.2	18.9	22.1
College	34.4	31.7	38.6	30.9
Post-college	29.6	27.7	27.4	28.0
Missing	2.8	0.66	0.43	0.11

Few women (11 exposed [0.50%] and 6 unexposed [0.55%]) and men (14 exposed [1.5%] and 6 unexposed [0.66%]) who answered the questions on sexual orientation identity selected the “prefer not to respond” option. Over 96% of respondents indicated that they were heterosexual. The distribution of sexual orientation and gender identity according to DES exposure status for women and men is shown in Table 2, along with ORs adjusted for birth year, education, and cohort. The OR for DES in relation to self-report of being nonheterosexual (gay/lesbian, bisexual or other) was 0.61 (95% CI = 0.40–0.92) for women and 1.4 (95% CI = 0.82–2.4) for men.

**Table 2:** Odds ratio (OR) and 95% confidence intervals (CI) for prenatal DES exposure and sexual orientation identity

	Women			Men		
	Exposed	Unexposed	OR (95% CI)^a^	Exposed	Unexposed	OR (95% CI)^a^
	*N* = 2220	*N* = 1086		*N* = 933	*N* = 915	
Sexual orientation identity						
Heterosexual	2142 (96.5)	1037 (95.5)	1.00	883 (94.6)	884 (96.6)	1.00
Nonheterosexual	67 (3.0)	43 (4.0)	0.61 (0.40–0.92)	36 (3.9)	25 (2.7)	1.39 (0.82–2.37)
Gay or Lesbian	30 (1.4)	28 (2.6)	0.44 (0.25–0.76)	21 (2.3)	15 (1.6)	1.44 (0.72–2.85)
Bisexual	33 (1.5)	12 (1.1)	0.96 (0.48–1.92)	13 (1.4)	8 (0.87)	1.41 (0.57–3.49)
Other	4 (0.18)	3 (0.28)		2 (0.21)	2 (0.22)	
Preferred not to respond	11 (0.50)	6 (0.55)		14 (1.5)	6 (0.66)	
Gender identity						
Woman	2214 (99.7)	1085 (99.9)		1 (0.11)	0 (0.00)	
Man	1 (0.05)	0 (0.0)		930 (99.7)	910 (99.5)	
Other	1 (0.05)	0 (0.0)		1 (0.11)	1 (0.11)	
Preferred not to respond	4 (0.18)	1 (0.1)		1 (0.11)	4 (0.44)	

^a^Odds ratios (OR) are from either binary logistic regression (nonheterosexual vs. heterosexual (reference group) as the dependent variable and DES as an independent variable) or polytomous logistic regression models (gay or lesbian, bisexual vs. heterosexual (reference group) as the dependent variable and DES as an independent variable). Models also included birth year (continuous), education and cohort as independent variables

We assessed, compared with the unexposed, whether DES dose or gestational week at first DES exposure in the women and men, and VEC in the women were related to sexual orientation identity (Table 3). Only women exposed to a low DES dose showed a reduced OR of reporting being nonheterosexual compared with self-report of being heterosexual (0.33, 95% CI = 0.13–0.85), though this was based on a small number of women. In contrast, among men, the OR of report of being nonheterosexual compared with heterosexual for those exposed to a high DES dose almost reached statistical significance (1.8, 95% CI = 0.96–3.3). Among women, ORs appeared similar by gestational week at DES exposure. While the ORs varied by gestational week among the men, they were based on a small number of participants. ORs for DES exposure compared with no exposure in relation to nonheterosexuality were 0.75 (95% CI = 0.44–1.3) in women with a history of VEC and 0.55 (95% CI = 0.30–0.99) in those without such a history.

**Table 3:** Odds ratio (OR) and 95% confidence intervals (CI) for prenatal DES exposure and sexual orientation identity by DES dose, timing of DES exposure, and presence or absence of vaginal epithelial changes (VEC)

	Women				Men			
	Hetero- sexual	Non- heterosexual	OR	95% CI	Hetero- sexual	Non- heterosexual	OR	95% CI
DES dose^a^								
Unexposed	1037 (96.0)	43 (4.0)	1.00	–	722 (96.9)	23 (3.1)	1.00	–
Low dose	469 (99.0)	5 (1.0)	0.33	0.13–0.85	412 (98.1)	8 (1.9)	0.68	0.30–1.57
High dose	1537 (96.2)	61 (3.8)	0.81	0.54–1.21	369 (93.9)	24 (6.1)	1.79	0.96–3.33
Gestational age at first exposure^b^								
Unexposed	687 (95.4)	33 (4.6)	1.00	–	617 (97.3)	17 (2.7)	1.00	–
<= 7 weeks	480 (96.2)	19 (3.8)	0.64	0.35–1.17	248 (94.7)	14 (5.3)	1.48	0.66–3.30
8–14 weeks	678 (98.0)	14 (2.0)	0.44	0.23–0.84	256 (97.7)	6 (2.3)	0.98	0.37–2.61
15 + weeks	394 (97.5)	10 (2.5)	0.63	0.30–1.32	149 (98.7)	2 (1.3)	0.55	0.12–2.49
VEC^c^								
Unexposed	593 (95.8)	26 (4.2)	1.00	–	–	–	–	–
Exposed, no VEC	851 (97.5)	22 (2.5)	0.55	0.30–0.99	–	–	–	–
Exposed, VEC	923 (96.3)	35 (3.7)	0.75	0.44–1.28	–	–	–	–

^a^Analysis of dose excludes one of the WHS cohorts (Dartmouth)

^b^Analysis of gestational age at first exposure excludes WHS cohorts

^c^Analysis of VEC includes DESAD and Dieckmann cohorts only

We also examined associations of DES exposure separately for lesbian/gay orientation and bisexual orientation. Sexual orientation identity reported as “other” was not examined as very few women (4 exposed and 3 unexposed) and men (2 exposed and 2 unexposed) reported it. Among women, DES exposure was inversely associated with reporting a gay/lesbian identity; OR 0.44 (95% CI 0.25–0.76) but was not associated with a bisexual identity (OR 0.96, 95% CI = 0.48–1.9) (Table 2). Among men, the ORs were elevated for DES in relation to reporting a gay identity (OR 1.4, 95% CI = 0.72–2.9) or bisexual identity (OR 1.4, 95% CI = 0.57–3.5), although confidence intervals were wide and included 1.0. The numbers of individuals in specific categories of sexual orientation identity were too small to evaluate possible associations with dose and timing of DES in women and men, or with the presence/absence of VEC in women.

Only two women, both DES exposed, and 3 men (2 exposed and 1 unexposed) reported gender identity that did not conform with the sex they were assigned at birth. Five DES-exposed persons and five unexposed preferred not to answer the question on gender identity.

Finally, we assessed the concordance of sexual orientation identity reported on the 2016 questionnaire with the previous report of adult sexual partners on the 1994 questionnaire. Of 2986 women who reported only opposite sex partners on the 1994 questionnaire, 30 (1%) reported bisexual, gay/lesbian or other on the 2016 questionnaire. Among men, the corresponding percent was 15 (0.88%) of 1704. In addition, we recomputed the OR for DES in relation to same-sex partners in the subset who also responded to the sexual orientation identity question in 2016. In this subset, the OR for DES in relation to same-sex partners was 0.65 (0.43–0.97) in women and 1.3 (0.74–2.4) in men.

## Discussion

The NCI combined cohorts of women and men represent one of the only human studies allowing evaluation of the influence of a potent synthetic estrogen on early fetal development in a population with documented DES exposure status. Brain organization that occurs in the prenatal period may influence an individual’s sexual and gender identities, with one hypothesis that variance in exposure to gonadal hormones may mediate this influence, along with genes and maternal factors (Fisher et al., 2018; Roselli, 2018), including maternal immune response (Bogaert et al., 2018).

We found that women exposed in utero to DES were significantly less likely to report being gay/lesbian, but DES exposure was not associated with bisexuality. The association with gay/lesbian orientation was strongest for exposure to a low cumulative dose of DES. We observed no consistent patterns of association between timing of first gestational DES exposure and sexual orientation identity among exposed women. The current results agree with those from our earlier study (Titus-Ernstoff et al., 2003), which indicated that prenatally DES-exposed women were slightly more likely than unexposed women to have ever married and were less likely to report sexual partners of the same sex.

In contrast to the findings for women, DES-exposed men were more likely to report being gay or bisexual; however, this estimate was imprecise likely owing to the men’s cohort being smaller than the women’s cohort. The findings in men agree with our previous study of sexual partners, in which the OR was elevated for same-sex partners, but confidence limits included 1.0 (Titus-Ernstoff et al., 2003). In one earlier report on prenatal DES exposure, exposed men were less likely than unexposed men to be married or living as married (Beral & Colwell, 1981), while two larger studies, including an analysis of 494 men in the Dieckmann randomized trial cohort, found no significant association between DES and ever having been married (Vessey, Fairweather, Norman-Smith, & Buckley, 1983; Wilcox, Baird, Weinberg, Hornsby, & Herbst, 1995). Taken together, our findings for the relation between prenatal DES exposure and sexual orientation identity in women and men are consistent with DES not acting as a masculinizing agent.

There were too few women or men reporting a gender identity different from that assigned at birth to analyze potential effects of prenatal DES exposure, but this suggests that any effect would be small.

Although nondifferential misclassification from underreporting of nonheterosexuality stemming from social stigmatization of homosexuality cannot be ruled out, it seems unlikely. The proportion of women and men reporting being LGBT (“Do you, personally, identify as lesbian, gay, bisexual or transgender?”) in a recent large Gallop poll (Newport, 2018) conducted in a random sample of Americans born from 1946 to 1964, approximately the birth cohort in our study, was lower (2.4%) than in our study (3.3% overall). Furthermore, the percentage of participants who did not respond or indicated they preferred not to respond was very low.

A limitation of our study was the restriction of the analysis to those who answered the 2016 questionnaire. Whether participant loss over the 30 years of follow-up was related to both sexual orientation/gender identity and DES exposure status is unknown. However, an association, whether positive or inverse, would be exaggerated only if participant loss to follow-up was also related to DES exposure status. It is also possible that reporting of sexual orientation and gender identity among the DES exposed was influenced by our previous publication indicating effects in women (Titus-Ernstoff, et al., 2003), but this seems unlikely, as concordance between 1994 and 2016 responses to the sexual orientation questions was extremely high. In addition, human sexuality is markedly complex, and our data addressed only one biological mechanism regarding exposure to exogenous hormones. As complicated as the etiology of sexual orientation is, the etiology of gender identity is even more elusive.

The rough approximation of sexual orientation and gender identity in our study may have limited our ability to capture the constructs being measured and thoroughly examine these characteristics with regard to DES exposure. Any random misclassification could have resulted in biasing our findings to the null. Although possible, it seems unlikely that our measures resulted in selectively underestimating the prevalence of nonheterosexual orientation in the DES-exposed women only. Although our study was large and has documented DES exposure status, it has limited power to assess associations with rare outcomes such as transgender identity. Despite limitations, these are unique data on a group of individuals exposed to high doses of synthetic estrogen during a particularly important development period.

In summary, the findings showed that women who were prenatally exposed to DES were significantly less likely to report being lesbian or bisexual. In contrast, while men who were prenatally exposed to DES were somewhat more likely to report being gay or bisexual, the estimate was very imprecise and compatible with chance. Finally, very few individuals reported currently thinking of themselves as a gender different from that assigned at birth in either the exposed or unexposed group.
